# The complexities of communication at hospital discharge of older patients: a qualitative study of healthcare professionals’ views

**DOI:** 10.1186/s12913-023-10192-5

**Published:** 2023-11-06

**Authors:** Henrik Cam, Björn Wennlöf, Ulrika Gillespie, Kristin Franzon, Elisabet I. Nielsen, Mia Ling, Karl-Johan Lindner, Thomas Gerardus Hendrik Kempen, Sofia Kälvemark Sporrong

**Affiliations:** 1 Hospital Pharmacy Department, Uppsala University Hospital, Uppsala, Sweden; 2 Department of Pharmacy, Uppsala University, Uppsala, Sweden; 3 Centre for Clinical Research, Uppsala University, Västmanland County Hospital, Västerås, Sweden; 4 Närvården Viksäng-Irsta, Region Västmanland, Västerås, Sweden; 5 Department of Public Health and Caring Sciences, Uppsala University, Uppsala, Sweden; 6 Department of Pharmacy, Region Västmanland, Västerås, Sweden; 7 Utrecht Institute for Pharmaceutical Sciences, Utrecht University, Utrecht, the Netherlands; 8 Department of Pharmacy, University of Copenhagen, Copenhagen, Denmark

**Keywords:** Aged, Continuity of patient care, Community health services, Health information exchange, Qualitative research, Primary healthcare, Patient safety

## Abstract

**Background:**

Hospital discharge of older patients is a high-risk situation in terms of patient safety. Due to the fragmentation of the healthcare system, communication and coordination between stakeholders are required at discharge. The aim of this study was to explore communication in general and medication information transfer in particular at hospital discharge of older patients from the perspective of healthcare professionals (HCPs) across different organisations within the healthcare system.

**Methods:**

We conducted a qualitative study using focus group and individual or group interviews with HCPs (physicians, nurses and pharmacists) across different healthcare organisations in Sweden. Data were collected from September to October 2021. A semi-structured interview guide including questions on current medication communication practices, possible improvements and feedback on suggestions for alternative processes was used. The data were analysed thematically, guided by the systematic text condensation method.

**Results:**

In total, four focus group and three semi-structured interviews were conducted with 23 HCPs. Three main themes were identified: 1) *Support systems that help and hinder* describes the use of support systems in the discharge process to compensate for the fragmentation of the healthcare system and the impact of these systems on HCPs’ communication; 2) *Communication between two separate worlds* depicts the difficulties in communication experienced by HCPs in different healthcare organisations and how they cope with them; and 3) *The large number of medically complex patients disrupts the communication* reveals how the highly pressurised healthcare system impacts on HCPs’ communication at hospital discharge.

**Conclusions:**

Communication at hospital discharge is hindered by the fragmented, highly pressurised healthcare system. HCPs are at risk of moral distress when coping with communication difficulties. Improved communication methods at hospital discharge are needed for the benefit of both patients and HCPs.

**Supplementary Information:**

The online version contains supplementary material available at 10.1186/s12913-023-10192-5.

## Background

The world population is ageing [1]. Older people have a higher disease burden and are twice as likely to require hospitalisation compared to middle-aged adults [2]. Providing healthcare to older people often involves numerous healthcare professionals within different healthcare organisations [3]. The coordination of care between the actors involved is challenging due to both structural and funding-based barriers [4, 5]. Transition of care is defined as different points in the care process where a patient moves to, or returns from, one healthcare setting to another, for example when a patient is discharged from a hospital ward to the home [6]. The hospital discharge process requires communication and coordination amongst stakeholders within the hospital and between the hospital and the primary healthcare organisations involved [7]. Evidence relates a deficient hospital discharge process to patient safety issues, such as increased medication-related harm [8] and greater healthcare utilisation [9].

### Medications in relation to hospital discharge

Medication usage is increasing amongst older patients, which is linked to a higher risk of drug-related problems (DRPs), such as adverse drug reactions, inappropriate prescribing and poor adherence [10, 11]. DRPs account for 9–15% of hospitalisations amongst older people [12, 13]. Older patients who are discharged from hospital are known to be prone to DRPs after discharge [14, 15]. Parekh et al*.* demonstrated that 37% of older patients experienced a DRP within eight weeks after hospital discharge, around half of which were potentially preventable [14]. Specific DRPs related to hospital discharge include medication continuity errors [16], inadequate follow-up after discharge [17], medication reconciliation errors [18] and insufficient patient involvement, resulting in patient confusion [19].

Nearly all hospitalised older patients experience at least one medication change that continues after hospital discharge, where the mean number is 3–4 changes [20–22]. The timely transfer of the discharge summary with clear requests for follow-up is considered crucial for the next healthcare provider [23]. However, discharge summaries are frequently delayed, of deficient quality and/or incomplete regarding follow-up plans [24, 25].

The deficits in the quality of the discharge documentation is known to cause confusion among patient/informal caregivers and may for example result in decreased compliance to the treatment [26, 27]. Furthermore, older patients express challenges in retaining medication information from their hospitalisation, despite receiving structured medication counselling at discharge [28]. The cause for this situation has been connected to patients not being sufficiently prepared and involved in their discharge [29, 30]. These findings were also confirmed in an unpublished qualitative study with discharged older patients conducted by our research group (Cam H, Franzon K, Kempen TGH, Nielsen I E, Gustavsson L, Moosavi E, et al: Involvement of older hospitalised patients in medication decisions: A naturalistic observation and interview study, in preparation).

### The Swedish context

The structure of care around older patients in Sweden is an example of healthcare that involves numerous organisations. In Sweden, regions and municipalities are the local governmental bodies responsible for providing healthcare services. Healthcare is divided into primary healthcare (e.g., nursing homes, home care services and primary healthcare centres) and inpatient/specialised healthcare (i.e. hospitals). The regions and municipalities have a shared responsibility for providing primary healthcare, while the regions are solely responsible for providing inpatient/specialised healthcare [31]. The municipalities and regions are tax-funded through two separate systems and are governed independently of each other.

The collaboration and communication required between the healthcare organisations involved in hospital discharge have led to the creation of legislation in this area (Table 1).

**Table 1:** Swedish legislation concerning communication at hospital discharge

**Requirements on collaboration between healthcare organisations **[34, 35]
1. The discharge planning should start as soon as the patient is admitted to hospital
2. If the patient is deemed to require healthcare services from her/his primary healthcare centre or municipal healthcare after discharge, information about the hospital admission, including an estimated discharge date, should be transferred to the healthcare organisations concerned
3. The recipients can then start planning for the provision of healthcare services after discharge
4. As soon as the hospital physicians assess the patient as ready for discharge, the healthcare organisations concerned should be notified
5. From that moment, the municipal healthcare organisations have three days to decide and prepare the healthcare services for which the municipality is responsible
6. If the time limit is exceeded, the municipality concerned is charged a fee of 880 EUR per day by the regional healthcare organisation
**Discharge communication requirements on the hospital **[18, 36]
• Two discharge summaries, written separately by a hospital physician (focusing on medical care) and a hospital nurse (focusing on nursing care) intended for the subsequent healthcare providers
• A patient friendly discharge letter written by the hospital physician containing a description of the hospitalisation course, the medication changes performed (including type of changes and why they were made) and the follow-up plans for the patient and/or informal caregivers
• An updated medication list

In Sweden, several IT-systems for electronic communication between the different healthcare organisations are used at discharge. The specific IT-system solutions for communication can be selected by the individual healthcare organisations, resulting in different IT-solutions and electronic health record (EHR)-systems within and across regions and municipalities.

Short-term nursing homes are a service provided by the municipalities, intended as a stopover for patients who are no longer in need of hospital care, but not fit enough to return home. The nurses at the short-term nursing homes are employed by the municipal healthcare authority, while the nursing home physicians are employed by the regional healthcare authority. The formal medical responsibility is shared between the short-term nursing home physician and the general practitioner (GP) at the primary healthcare centre where the patient is registered. The nursing home physicians are expected to treat all of the patients’ urgent medical issues, while the GP is required to deal with all non-urgent issues (e.g., prescribing non-urgent medications or continuing non-urgent investigations suggested by the hospital) [32].

New nursing roles have emerged to support hospital discharge. Hospital discharge nurses coordinate patient discharge at the ward and communicate with primary and municipal care as well as with informal caregivers. The discharge nurses are usually not involved in the everyday care of the patient at the ward. The corresponding nursing role also exists on the receiving side (primary healthcare centre discharge nurses). However, it is not mandatory for these roles to be present at either hospital wards or primary healthcare centres.

### Healthcare professionals’ perspective on communication at hospital discharge

Several previous qualitative studies have focused on the experience of the hospital discharge process in general [33–36] and on medication information in particular [37] among healthcare professionals (HCPs) of different professional categories. A common result is that the discharge process is complexas the healthcare organisation comprises numerous barriers to as well as facilitators of communication, which impact on HCPs’ ability to deliver safe hospital discharge for older patients [33–37]. Based on the findings from these previous studies, we wished to further explore communication at hospital discharge in a wider context.

The aim of this study was to explore communication in general and medication information transfer in particular at hospital discharge of older patients from the perspective of healthcare professionals (HCPs) across different organisations within the healthcare system.

## Methods

We conducted a qualitative study, primarily using focus groups [38] comprising HCPs from two regions (labelled as Region A and Region B) in Sweden. Subsequent semi-structured interviews [39] were performed to complement the data derived from the focus group interviews. The focus group and semi-structured interviews took place from September to October 2021. The study was approved by the Swedish Ethical Review Authority (Reg.no.: 2020–02734). Written informed consent was obtained from all participants prior to participation. The study is part of the Improved Medication information and Patient involvement At Care Transitions (IMPACT-care) research project, which aims to develop an intervention to improve medication safety and older patients’ experiences at hospital discharge [40].

### Study setting

**Fig. 1:**
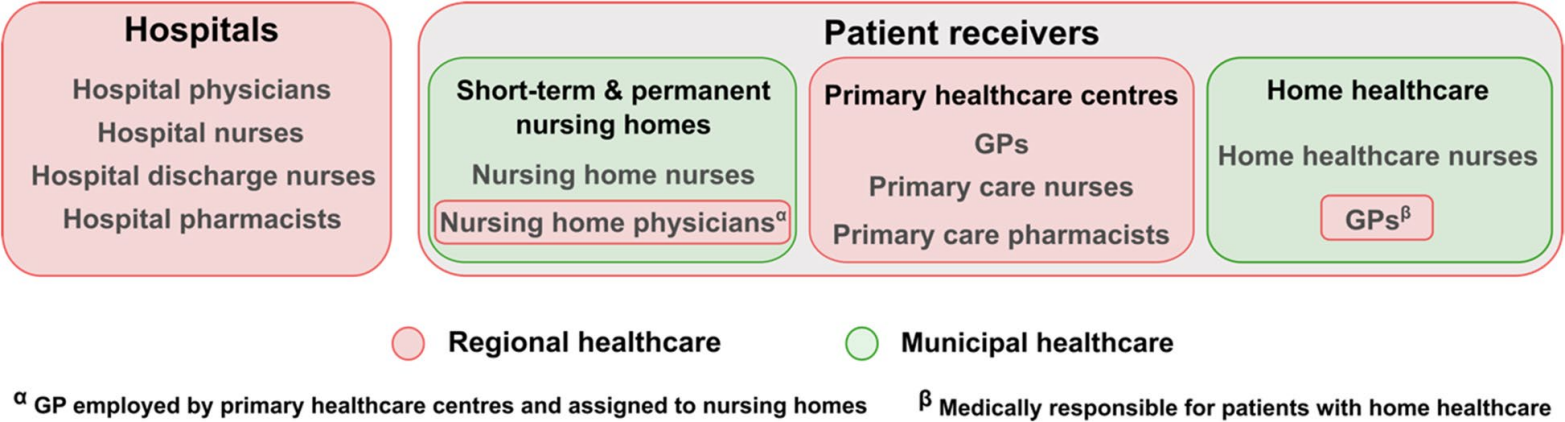
Schematic diagram illustrating relevant healthcare professionals involved in medication communication, grouped by the healthcare organisations they belong to (either under the responsibility of the region or the municipality). *GP* = general practitioner

Region A comprises eight municipalities and has a population of around 390,000 people, while Region B has 10 municipalities with a population of around 280,000 people [41]. This study includes the following healthcare organisations: hospitals, primary healthcare centres, short-term nursing homes, permanent nursing homes and home healthcare. The regions are responsible for the first two, while the municipalities are responsible for the rest (Fig. 1).

Hospitals and primary healthcare centres within the two regions where this study was conducted share a common EHR-system, thus HCPs in hospitals can access the records from HCPs in primary healthcare centres and vice versa (Table 2). At the time this study was conducted, a few municipalities had the same EHR-system as the hospitals and primary healthcare centres, while others did not. However, the information in the regional EHR was accessible to a limited extent to nurses employed by the municipality through the national external IT-system (National patient overview) [42]. The nursing home physicians have full access to the EHR, as they are employed by the regional healthcare organisation. In both regions, written discharge information is available to all HCPs in regional healthcare through the shared EHR. However, for GPs to be actively notified, the hospital physicians are required to send an electronic referral with a request for follow-up in addition to the discharge summaries (e.g., to request the GP to take responsibility for performing follow-up treatment) [43].

**Table 2:** IT-systems for communication about medications at hospital discharge used by HCPs in regional and municipality healthcare in this study

IT-system (software name)	Accessibility regional HCPs	Accessibility municipal HCPs	Comment
Regional EHR (Cosmic)	Full access^α^	Read-only access^β^ (through the national EHR)	A shared EHR is used between the hospital and primary healthcare centres in Region A and Region B
Municipal EHR (N/A^δ^)	No access^β^	Full access	Municipal HCPs only have full access to their own EHR
National EHR (National patient overview)	Read-only access	Read-only access	An external national system to access patient information regardless of healthcare organisation. Information from the two regional EHRs is automatically transferred to the system. Information from the municipal EHRs is not transferred
Electronic communication system (Cosmic-Link & Prator)	Full access	Full access	An electronic communication system for HCPs belonging to different healthcare organisations, enabling them to collaborate and plan patient care
Multidose drug dispensing prescription (Pascal)	Full access	Full access	A national system for physicians to prescribe medications in cases where a patient has multidose drug dispensing. The medication information in this system is not automatically synchronised with the local EHR

*EHR* electronic health records, *HCPs* healthcare professionals

^**α**^ Writing, reading and saving information. ^β^ Full access if the municipality uses the same EHR-system as the regional healthcare authority. ^δ^ Numerous types of software in use depending on the municipality

### Participants

The following professional categories were invited to participate in this study: physicians from hospitals and primary healthcare centres; nurses from hospitals, primary healthcare centres and municipal care; and clinical pharmacists from hospitals and primary healthcare centres. The hospital-based HCPs were recruited from medical wards. The participants had to be involved in medication-related patient care at hospital discharge. A purposeful sampling approach [38] was adopted to obtain a heterogeneous sample in terms of gender, experience and workplace. Potential participants were identified through the researchers’ professional networks. In the event of potential participants declining, this method was complemented with snowball sampling by asking if they could recommend anyone with the appropriate background who might be interested in participation. They were contacted by the researchers through email, telephone, or both, with the request to participate in a focus group session or individual interview. The participants were not offered any compensation but received a surprise in the form of a 300 SEK (28 EUR) gift card on conclusion of the study.

### Data collection

The participants were divided into focus groups based on region and municipalities within the region. The hospital-based participants were placed in separate focus groups, while the primary and municipal care-based participants were mixed in focus groups. This division was made to separately capture the perspectives of those discharging the patient and those receiving the patient. Additional individual or group interviews were conducted with participants who were unable to attend the focus groups as intended.

Three semi-structured interview guides (Additional file 1) were developed, one for the focus groups comprising of hospital-based HCPs, one for the focus groups with patient receivers and the third for the interviews. The interview guides were derived from the literature [37], from discussions within our research team consisting of multidisciplinary clinicians, researchers and patient representatives, and from an unpublished earlier study on older patients’ experiences of medication communication at discharge (Cam H, Franzon K, Kempen TGH, Nielsen I E, Gustavsson L, Moosavi E, et al: Involvement of older hospitalised patients in medication decisions: A naturalistic observation and interview study, in preparation). The semi-structured guides contained open-ended questions, used as starting points for discussions. Follow-up questions were posed when relevant. Three main topics were outlined: 1) Current communication about medications at hospital discharge and associated barriers and facilitators; 2) Suggested improvements to communication about medications at hospital discharge; 3) Feedback on suggested new discharge processes. Four focus group sessions were conducted, the first of which was considered a pilot test for the interview guides. The data collected at this session were included in the analysis. Modifications to the interview guides were made iteratively [44] as the study progressed.

The focus groups were led by a moderator of female gender (SKS), a social scientist with an extensive background in health-related qualitative research and previous experience of moderating focus groups. She introduced herself to the participants as a university lecturer. One co-moderator of male gender (HC or BW) was present at each focus group session and responsible for taking field notes and assisting in asking follow-up questions on the topics discussed. Both co-moderators were clinicians (HC a clinical pharmacist and BW a resident physician in family medicine) trained in qualitative research methodology. They introduced themselves to the participants as researchers. The focus group sessions were held in a hospital meeting room. The semi-structured interviews were conducted by one of the first two authors (HC or BW) in a room at the participant’s healthcare unit. The focus group and interview sessions were audio-recorded and transcribed verbatim. There were few prior professional relationships between the (co-)moderators or interviewer and the participants.

### Data analysis

The transcripts and field notes were analysed thematically, guided by the systematic text condensation method described by Malterud [45]. The Nvivo software program version 1.5 was used. Consensus meetings were held on a regular basis and an inductive approach was adopted throughout the data analysis, which was divided into the following steps:

1. HC and BW thoroughly read all transcripts to gain a general understanding of the data.
2. One transcript from a hospital focus group and one from a patient receivers group were individually coded by HC, BW, UG (female clinical pharmacist and senior researcher), KF (female physician specialised in geriatrics), EN (female pharmacist and senior researcher) and SKS.
3. A consensus meeting attended by HC, BW, UG, KF, EN and SKS was held. An initial coding scheme and overarching themes were developed.
4. Individual coding of all transcripts was conducted by BW and HC in accordance with the initial coding scheme. Regular consensus meetings between the two authors were held. The coding scheme was developed as new codes, sub-themes and main themes emerged from the data.
5. An audit of the coding was conducted. The coded transcripts were divided between UG, KF, EN and SKS and read to confirm that no meaning units were missed and that the coding was logical.
6. A consensus meeting between HC, BW, UG, KF, EN and SKS was held. The coding scheme was adjusted, and the content of the codes was refined.
7. Condensation of the content of the codes was performed by HC and BW. These authors reconceptualised the condensed data into new main themes and sub-themes with accompanying descriptions and concepts.
8. A final consensus meeting was held attended by HC, BW, UG, KF, EN and SKS to verify that the main themes and sub-themes were firmly grounded in the original data.

Although communication between HCPs in different organisations is complex and multidirectional, we have termed the hospitals ‘*information senders’* and the patient receiver organisations as ‘*information receivers’* to simplify the reporting in this study.

## Results

Twenty-six HCPs were invited to participate in the focus groups and twenty-four agreed. However, five did not attend the focus group sessions due to pressing work responsibilities. Four of these were invited and agreed to attend the complementary interview sessions. In total, 23 HCPs took part in the study (Table 3). The focus group sessions lasted between 95 and 120 min. An additional three interviews were conducted with four participants, each of which took from 50–55 min.

**Table 3:** Profession and gender of participants in the focus groups and interviews

Focus group (patient receivers)			Focus group (hospitals)			Semi-structured interviews	
Profession	Group 1 (*n* = 4)	Group 2 (*n* = 6)	Profession	Group 3 (*n* = 6)	Group 4 (*n* = 3)	Profession	3 interviews (*n* = 4)
GP	♀	♀	Hospital physician	♀ + ♂	♀	Nursing home physician	♂
Nursing home physician	-	♂	Hospital nurse	♀ + ♀	♀	Hospital discharge nurse	♀ + ♀
Primary care nurse	♀	♀	Hospital discharge nurse	♀		Nursing home nurse	♀
Home healthcare nurse	♀	♀	Hospital pharmacist	♀	♀		
Nursing home nurse	-	♀					
Primary care pharmacist	♀	♀					

*GP* general practitioner

**Fig. 2:**
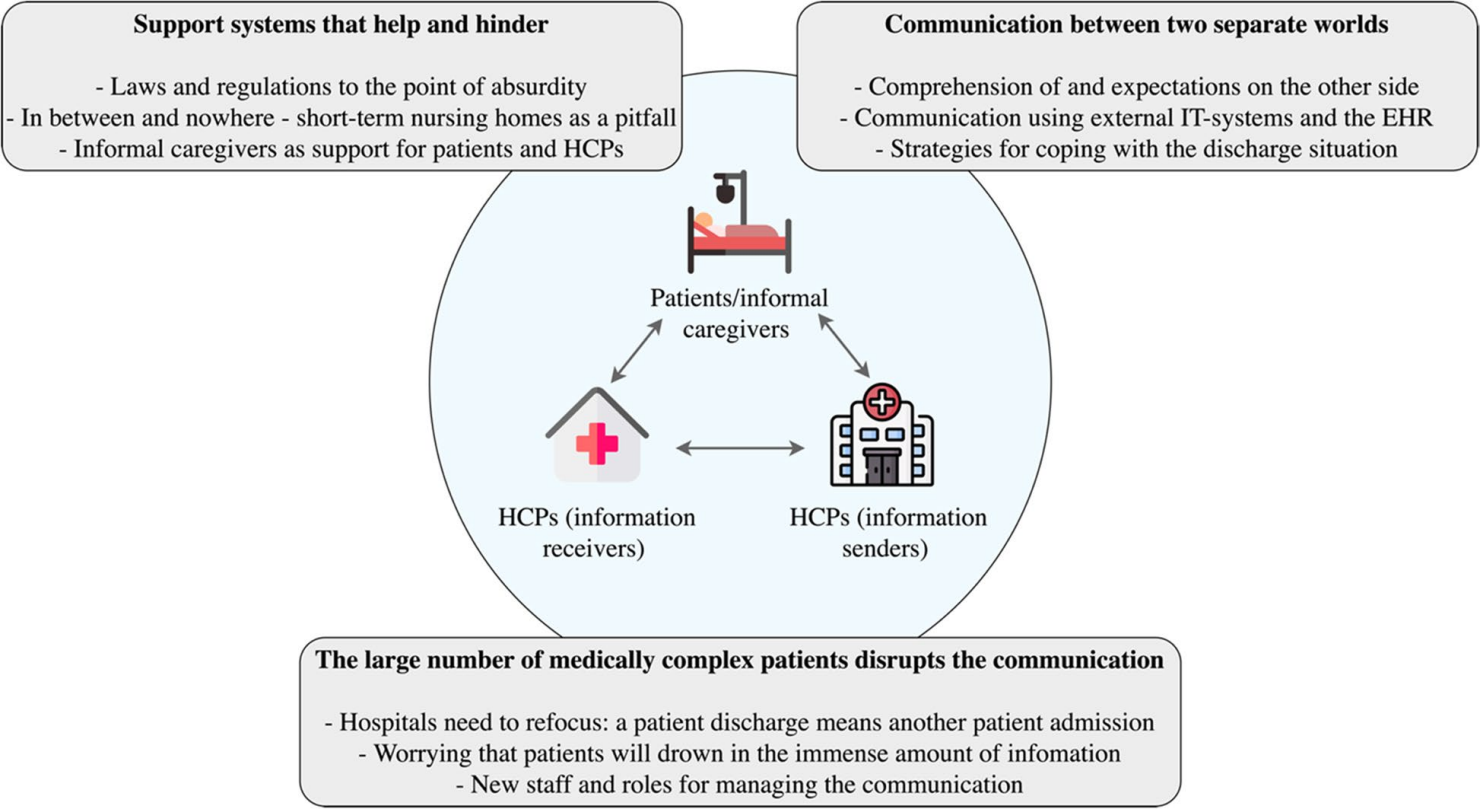
The three main themes, along with their sub-themes, that influence medication communication at hospital discharge. These themes pertain to the three primary actors: patients/informal caregivers, HCPs on the information receiving side, and HCPs on the information sending side. *EHR* = electronic health records, *HCP* = healthcare professional

The analysis resulted in three main themes: 1) Support systems that help and hinder, 2) Communication between two separate worlds and 3) The large number of medically complex patients disrupts the communication, all with several sub-themes (Fig. 2).

### Support systems that help and hinder

Regulations, short-term nursing homes and informal caregivers were frequently mentioned as supporting the HCPs in the management of communication at discharge in an effective and appropriate way. However, the support was sometimes perceived as too inflexible or unreliable to lead to an optimal patient outcome.

This theme comprised the following three sub-themes: 1) Laws and regulations to the point of absurdity, 2) Informal caregivers as support for patients and HCPs, and 3) In between and nowhere – short-term nursing homes as a pitfall.

#### Laws and regulations to the point of absurdity

The participants’ general perception was that the discharge process has become too complex for the communication to progress smoothly and that too many separate healthcare organisations are involved. In addition, communication between HCPs often does not primarily focus on the patient, but on who is responsible for what, including who is paying and/or getting paid for the care provided. The HCPs on both sides claimed that the legislation pertaining to hospital discharge has resulted in a rigid and inflexible system, generating friction between HCPs and diminishing the focus on patients’ needs. They were distressed about the fact that the patients were increasingly viewed as “products” that one has to squeeze through the discharge process.

*“When I think about the discharge process, it makes me think about the communication between lots of different participants. So many are involved. Sometimes you don’t discuss the patient that much /…/ But there are so many practicalities all the time. There’s a lot of discussion about responsibility. Who should do what and at what stage /…/ And no one really knows what other people’s responsibilities are.”* (Home care nurse 1, female, focus group 1)

A majority of the participants found the bureaucracy concerning the discharge process too complicated. For example, primary healthcare centres are designated by law to coordinate patient discharge. However, as they are not involved in patient care during the hospitalisation, they do not have first-hand knowledge about patients’ current health status and care needs. The primary healthcare centres were described as “middle-men” through whom the communication between hospital and municipality must go. Participants from the sending side experienced this as ineffective and unsafe due to the substantial risk of information loss when not in direct contact with the next responsible healthcare provider. GPs found themselves formally responsible for patients they may never have met. They experienced a loss of control due to the complexity of the discharge process and described situations of being handed forms to fill in, without an opportunity to assess the situation and sometimes not even knowing what and why they are signing.

*“Sometimes I feel like a confused office clerk /…/ We often get such tasks: The hospital says now you should do this and then someone else says now you should do that. Then I try to do something in the middle, without really knowing how and why.”* (Primary care physician 1, female, focus group 1)

The nurses from the municipality claimed that they do not have any direct contact with the patient before discharge, as all contact is managed via the primary healthcare centre. This resulted in the preparation of care and support that often patients later declined after discharge. There was consensus among the participants that communication would be more effective if the system permitted the hospital and municipality to contact each other directly.

The privacy laws regarding patient information between healthcare organisations was another regulation that was highlighted as frustrating and a patient safety issue. The nurses from the municipality suspected that the regional healthcare HPCs lack insight into just how limited EHR access is in the municipalities.

*“We don’t see very much, I don’t think anyone understands that we never see the Cosmic medication list [the list in the EHR-system]. /…/ So we’re very dependent on getting the medication list when the patient is discharged /…/ Otherwise we only have the old list to go by.”* (Nursing home nurse 2, female, interview)

#### In between and nowhere – short-term nursing homes as a pitfall

The short-term nursing home participants repeatedly expressed the unclear role of short-term nursing homes in terms of who has medical responsibility for the patient. They described a major discrepancy between how the division of responsibility for the patient is supposed to function in theory and the reality, which led to frustration and lack of continuity of care for patients discharged to short-term nursing homes. According to the participants, in reality the nursing home physician is supposed to solve *all* medical issues including non-urgent ones while the patient is at the nursing home, despite being solely officially responsible for *urgent* medical issues and only has time allocated for this. The participants argued that this discrepancy negatively affected the follow-up of patients. The requests for follow-up are sent by the hospital physicians to the GPs and not to the nursing home physicians, risking planned follow-ups being missed.

*“I am responsible for the urgent issues. The primary healthcare centre where they are registered has formal responsibility for the patient /…/ but it becomes unreasonable /…/ I prescribe a lot of medications /…/ but the hospital may have forgotten to dispense them, then it becomes urgent. I must prescribe them. /…/ The hours that are allocated are only intended for urgent assessments. But that is not the case at all. What happens is that you take care of everything.”* (Nursing home physician 1, male, interview)

This discrepancy between theory and reality in terms of responsibility was also regarded as a risk factor by GPs. The difficulties related to follow-up of the patients’ medications after discharge to a short-term nursing home were highlighted as a risk factor. GPs described a risk of non-urgent matters in the follow-up request falling between the cracks. Both GPs and nursing home physicians stated that, in reality, the responsibility for processing the follow-up request lies with the nursing home physician as long as the patient is there. The GPs also claimed that there is no support system to actively notify them when patients leave the short-term nursing home and are thus their responsibility again. They wanted to be actively notified when the patient returns home from the short-term nursing home.

*“When they [patients] come from the short-term unit to home it’s even more difficult. Because then the follow-up request may be a month old. And it’s very rare that the nursing home physician sends a follow-up request to us. And regarding those patients, I think they really need to be identified. /…/ Because they are usually the most frail.”* (Primary care physician 1, female, focus group 1)

#### Informal caregivers as support for patients and HCPs

The participants generally considered it beneficial to involve informal caregivers at hospital discharge, especially if the patient is cognitively impaired. The hospital participants stressed that it must be difficult for patients to comprehend all the information provided at discharge. For them, the main reasons for contacting the informal caregivers were to gain an insight into how the patients had managed their life and medications at home prior to hospitalisation and to be confident that someone is there to take care of the patient after discharge.

*“It’s difficult when you see a cognitive impairment on the ward and the patient refuses to accept the help we think they need. Then it feels like the informal caregivers are very important, and we ask them – Have things worked out before? Because things might work out when they’re in their home environment and taking the tablets they’re used to taking even though they have a cognitive impairment.”* (Hospital nurse 2, female, focus group 3)

The participants pointed out that the informal caregivers’ attitude can vary. Some want to be involved and are eager to know everything that happened during hospitalisation, while others can be more distant. Despite the general opinion that involving informal caregivers is beneficial, problematic issues were also raised by participants. An example is when the informal caregivers’ opinions differ from those of HCPs or the patient regarding the amount of care support she/he will need after discharge.

*“There’s also a problem when informal caregivers and patients think two different things. /…/ Often the patient refuses any help whatsoever and their informal caregivers tell them something like – yes, but that won’t work.”* (Hospital discharge nurse 3, female, interview)

### Communication between two separate worlds

In our data, the senders and receivers appeared as two separate entities handling hospital discharges from their own perspective, with limited communication and insight about “the other side”. We found several examples of senders and receivers having conflicting and contradictory ways of working with the discharge process. Some of the HCPs’ key approaches to handling the discharge situation also emerged. This theme was based on the following three sub-themes: 1) Comprehension of and expectations on the other side, 2) Communication using external IT-systems and the EHR, and 3) Strategies for coping with the discharge situation.

#### Comprehension of and expectations on the other side

A common perception amongst the participants was that there is a lack of knowledge about responsibilities and undertakings of “the others” in the discharge process. Both sides claimed that they try to contact the other side early in the discharge process to facilitate the proactive preparation of the discharge, but there was a common, contradictory perception that the response from the other side is passive until the discharge becomes urgent. The hospital participants expected the receivers to be more proactive in the communication about and preparation of the discharge and to monitor more closely for any updates and information sent to them.

*“So, I can enter a patient in Prator [electronic communication system]. Two weeks later, when the patient’s care with us is finished and we /…/ issue a discharge note, that’s when they [the receivers] start planning. And that’s probably where the frustration starts. That they perhaps don’t recognise the need at an earlier stage and start to plan when we admit the patient.”* (Hospital discharge nurse 2, female, interview)

Likewise, the nurses on the receiving side frequently mentioned the need to closely monitor the patient’s hospitalisation course and information flow. They sometimes perceived the hospital discharge as rushed, stating that the information in the EHR was often unclear. This meant that they had to ask the sending side for clarification to facilitate patient discharge.

*“It’s often the case that you wish there could have been a little more foresight. /…/ But when someone is registered in Cosmic Link [electronic communication system], I can be active and ask – what sort of need does the patient have? What do you all think? What’s your opinion? What can we do to prepare something? It may be the case that you get no response at all, until boom!, now everything is ready and the patient is due to go home. Need help with this, that and the other. Can the patient go home today? Transport ordered for 2 PM. And then everything falls into your lap.”* (Home care nurse 1, female, focus group 1)

#### Communication using external IT-systems and the EHR

During the discussions it was apparent that HCPs involved in the discharge process generally communicate by means of several different IT-systems. Physicians on both sides (as opposed to nurses) rarely seemed to communicate directly with each other regarding a specific patient at discharge. This lack of direct communication was perceived as unsatisfactory by the receiving physicians, firstly because the referrals with follow-up requests by the sender may be delayed, and secondly because it can take time before the referral is read by the receiver. The receiving physicians expressed a wish for direct contact with the hospital physician prior to discharge, especially to discuss complex patients with multimorbidity or those who are at a palliative stage. Some of the hospital physicians also wished for direct communication.

*“Sometimes you would wish that we were contacted more directly when very ill patients are discharged. It’s unsatisfactory that medical communication takes place via follow-up requests with long lead times.”* (Primary care physician 1, female, focus group 1)

Participants frequently stated that the IT-systems were frustrating and limiting. A common reason for frustration was that some systems were not integrated in the EHR system, which made the information fragmented and difficult to interpret. The IT-system used for prescribing medications for multi-dose drug dispensing (Pascal) was frequently mentioned as such a system, generating discrepancies in the patients’ medication lists.

*”It’s confusing with two systems and if you prescribe in Cosmic [EHR-system] it will appear in Pascal [multidose drug dispensing prescribing-system] but if you prescribe in Pascal it will not automatically appear in Cosmic.”* (Nursing home physician 2, male, focus group 2)

Nevertheless, communication by means of IT-systems was recognised as an absolute necessity. A common view amongst the participants was that the communication between the hospital and primary healthcare centres has improved significantly since the introduction of a common EHR. The physicians from both sides viewed the discharge summary as an important document for obtaining an overview of the patient’s hospitalisation trajectory and the medication changes made. The hospital physicians highlighted the importance of writing this summary but added that it was difficult to write it in a way that fulfilled the needs of different HCPs. However, the receiving physicians stated that it is too difficult to find the relevant information in the discharge summaries. In addition, they wanted more explicit information on reasons for medication changes and plans for follow-up of newly prescribed medications. They mentioned that the care following hospitalisation is dependent on how well the discharge summary is written, which is problematic, as there is sometimes a delay before such a summary is even written.

*“There are uncertainties about where this medication dose originated and [why the patient] went home with this dose, or [why] this medication at all, and so you start to investigate. Well, it's great to have access to the medical records but then you end up with a barrow load of notes /…/ what we want to know is, OK there was a [medication] change, why and how it should be followed up and then you're often thinking – no, we don't know – so then you have to guess why it turned out like that.”* (Primary care physician 2, female, focus group 2)

The GPs and primary care pharmacists frequently found errors in the written medication information at discharge, especially in the medication list. The home care and nursing home nurses highlighted the importance of access to the medication list before discharge (via the GP) to prepare the supply of medications for the patient. However, they reported that they had never reflected on the possibility that the information in the medication list could be incorrect.

*“But of course, the medication list is still something you can't completely trust. So I always feel uncertain after opening a list of 20 medications for a patient who has just been discharged. /…/ It doesn't automatically correspond with the list that will be included in the patient discharge summary, mind you, a medication list is not always included in the discharge notes. /…/ It never feels particularly safe to send one [the medication list to the nurses in the municipality] when you haven't met the patient, who has been in hospital for a long time and so the medication list is completely different, and then of course the home care service wants a medication list, so we send it and hope that it's correct.”* (Primary care physician 1, female, focus group 1)

*“We often run into problems with the medication list. I have never thought about it from the point of view that as a physician you might feel uncertain, I just want a list.”* (Home care nurse 1, female, focus group 1)

If a hospital pharmacist had reviewed the medication at discharge and written a note in the EHR, the participants on the receiving side felt that they could trust the discharge medication list and written documentation about medication changes performed during hospitalisation.

*“In general, I think that the medication list is better now. It's much safer now that you can access and read the [hospital] pharmacist's notes.”* (Primary care physician 1, female, focus group 1)

#### Strategies for coping with the discharge situation

We identified three ways of coping with the obstacles to enable effective communication at hospital discharge: 1) *Smoothening the process by moderating the patients’ autonomy and self-care decisions.* During hospitalisation the hospital-based HCPs assume responsibility for medications, but when the patients return home, they are expected to manage the medications themselves, otherwise home healthcare is necessary. Participants on the sending side repeatedly mentioned that as soon as they notice a risk of non-compliance, they try to motivate the patients to accept home healthcare. The participants pointed out that some patients are readmitted to hospital due to self-care failure after discharge. The importance of making a thorough evaluation of the patients’ capacity for self-care and administering their own medications was highlighted. In general, participants who adhered to this coping strategy seemed to be uneasy with the patient’s self-care ability after discharge. They do not trust the ability of patients to know what is best for themselves and instead try to persuade them to accept what the HCP thinks is the safest way to proceed.

*“We try to tie different threads together, to make things work as well for the patient at home as in hospital /…/ after discharge some patients refuse the help that we have fought so hard for them to get /…/ we have tried to persuade the patient /…/ and yet soon after they return to their home environment, they close the door on the homecare staff and refuse to accept any help. And they get on with their own lives.”* (Hospital discharge nurse 1, female, focus group 3)

Participants on the receiving side perceived that home healthcare is sometimes initiated too quickly instead of, for example, helping patients to manage medications themselves. It was common for patients to cancel the home healthcare service after hospital discharge.

*“Then when you make this first home visit, for example – the patient hasn’t been given any information and doesn’t understand. And it’s quite often the case that when you visit the patient at home, it’s – no, I can do this myself, I want to do this myself. So then you just have to cancel everything.”* (Home care nurse 1, female, focus group 1)

2) *Bypassing the rules and official routines.* The HCPs who adhered to this strategy did not trust that the rules would ensure a successful discharge. In order to make the situation better and smoother for patients, some HCPs made a larger than expected effort to find ways to bridge the gaps in the system (e.g., sending HCPs to contact municipal care directly instead of through the patient’s primary care centre).

*“The healthcare centres have different levels of commitment /…/ sometimes you can’t get hold of them, you don’t get any confirmation – Have you seen this? Will you share medical responsibility? Will this patient get any help now? – And sometimes you decide to mediate, you call the homecare yourself.”* (Hospital nurse 1, female, focus group 3)

Participants on both sides, especially nurses, often mentioned that they phoned the other side to confirm or clarify issues, despite being aware that officially, communication should take place in written form via the electronic communication system. A common view was that if you want to resolve an issue quickly, the official communication system could not be trusted and it was better to phone.

*“If a nurse writes notes in the health records, the home care doesn’t have access to that information. Because it’s covered under the GDPR [general data protection regulation] and the Patient Data Act and so on, /…/ you have to use this [shows a mobile phone] and then this [shows Post-It Notes] and then you have to send a fax.”* (Home care nurse 1, female, focus group 1)

3) *Adhering to the rules and official routines and accepting that discharge may fail*. The HCPs following this strategy either trusted the system to work or accepted that it could fail. One issue highlighted by hospital participants was a sense of powerlessness in deciding about whether patients need home healthcare after discharge. They perceived that the decision is taken by the municipality in discussion with the patient/informal caregivers.

*“Because as a physician I can see quite easily that – Well, this patient, he can’t go home. This patient has to go to a short-term nursing home – or it will never be viable. But I’m not allowed to communicate that. Instead, the communication should always be only between the municipality and the patient. Otherwise, it’ll become almost like a fight over prestige /…/ I have no power in that situation.”* (Hospital physician 3, female, focus group 4)

HCPs from the primary care centres felt frustrated when the hospitals decided to discharge a patient, even though the primary care centre staff were not ready to receive her/him. The receiving side felt that they had to accept the situation, without the hospital being made accountable.

To illustrate instances when divergent coping strategies adopted by the HCPs can lead to communication problems, the example of when hospital physicians send an electronic referral to the GPs with requests for follow-up at discharge is highlighted in Table 4. Hospital physicians described that they tend to employ the second coping strategy and assumed that the receiving GPs employ the same strategy when arranging follow-up after discharge, while the receiving GPs tended to adopt the third strategy due to pressing work responsibilities.

**Table 4:** An illustrative example of how divergent coping strategies, adopted by HCPs and described in the sub-theme “Strategies for coping with the discharge situation”, can cause communication problems. This example highlights when electronic referral with requests for follow-up from hospital physicians to GPs at hospital discharge are used  as a one-way communication tool

HCP	Intended way of handling a referral with request for follow-up	Actual way of handling a referral with request for follow-up to cope with the discharge process	Quote
**Sender (hospital physician)**	A referral with precise and specific requests and clearly defined follow-up plans or recommendation of follow-up of specific problems	A referral with broad and somewhat unclear requests, attempting to ask the receiving GP to take responsibility for multiple issues (*coping strategy 2*), assuming that the receiver also adheres to this strategy	*“We can send off a referral with a follow-up request to primary care and say they should follow up on something specific, such as adjusting blood pressure medication or similar. But it feels as if it can have several purposes. You think that there’s someone who is addressing the situation, now and then I say that – this is what we have done and our thoughts about it – and then it becomes like some indirect follow-up of the other problems as well.”* (Hospital physician 2, male, focus group 3)
**Receiver (GP)**	Accepting the referral and assessing the need for the requested actions	Declining the referral because of lack of clarity (*coping strategy 3*)	*“Quite often we receive purely general follow-up requests. We’re not so keen on that. Because not everyone needs to be followed up. You’d like someone to think – Does that really need to be followed up?”* (Primary care physician 1, female, focus group 1)

*GP* General practitioner, *HCP* Healthcare professional

### The large number of medically complex patients disrupts the communication

The sheer number of medically complex patients contributed to the difficulties in communication at hospital discharge. This theme comprised the following three sub-themes: 1) Hospitals need to refocus: a patient discharge means another patient admission, 2) Worrying that patients will drown in the immense amount of information, and 3) New staff and roles for managing the communication.

#### Hospitals need to refocus: a patient discharge means another patient admission

The hospital participants frequently mentioned that discharge is followed by admission, thus relieving the emergency department. They therefore felt compelled to discharge patients to receive new ones. It was also pointed out that this is sometimes the reason for mistakes, especially when the discharge has been too rushed, which results in patients being readmitted to hospital.

*“We prioritise discharges in the morning. That is, we try to empty the beds so that we can admit new patients. Because the pressure is so high now, we have very short hospitalisations but then we get readmitted patients instead.”* (Hospital discharge nurse 3, female, focus group 3)

The hospital participants’ perception was that the discharge date can be decided suddenly, even on the same day as the patient leaves. Discharge day was described as a hectic time filled with practical activities for the patients that the hospital-based HCPs had to deal with and coordinate.

*“Yes, arrange transport home, talk to informal caregivers, talk to the nursing home. And talk to the homecare if they have it. Coordinate on the ward so that everyone knows that this patient is going home, what time they’re going home at, that there is someone coming … The [patient] room has to be cleaned, you have someone else waiting for the bed. Has everyone played their part to make sure this patient can get home safely? And at a certain point I often feel pressed for time. Has the patient received his/her medication list? Has the patient received his/her medications? Have we checked the skin? Does the skin have any wounds? Have all peripheral venous catheters been removed?”* (Hospital nurse 1, female, focus group 3)

Consequently, the hospital physicians mentioned that they sometimes conduct the discharge consultation with the patient in the common area at the ward, despite their opinion that it should be held in a private patient room. The physicians argued that it is difficult to plan a set time for the discharge consultations due to the erratic environment on the ward. Unexpected urgent events such as stroke or cardiac arrest calls can occur at any time. From the hospital physicians’ perspective, the optimal situation would be to give all information to the patient on the day prior to discharge, in order to avoid all the stress and pressure on the day of discharge.

#### Worrying that patients will drown in the immense amount of information

The immense amount of information given to the patient at discharge can make it difficult for her/him to comprehend it all. The hospital participants stated that the time for informing and ensuring that the patient understands is limited. They were aware that patients are not able to remember everything they say. Importance was instead attributed to the written information given to the patient at discharge, namely the patient friendly discharge letter, and how best to write it so that the patient can interpret it correctly. The letter was seen as something to be referred to after discharge if anything was unclear to the patient or informal caregiver.

*“The important info is in the patient friendly discharge letter from the physicians. /…/ Because I’ve heard it from the patients when I’ve rung them up after they’ve been at home for a while. They don’t remember. Then the most important info should be on the discharge letter, if there is anything special they have to do, or what medication changes have been made.”* (Hospital pharmacist 1, female, focus group 3)

The participants on the receiving side confirmed these worries about the patients’ lack of understanding of the information at discharge. They frequently experienced that patients have many questions after discharge and are confused about all information they have received.

*“When they contact us, we can explain what has been done in the hospital. It takes up most of the time allocated to patient consultations, what was actually done by them [the hospital], I don’t know how it should be followed-up so I want to meet you, because I want to talk to you about what happened.”* (Primary care physician 2, female, focus group 2)

#### New staff and roles for managing the communication

Participants stated that high staff turnover, especially nurses, within all healthcare organisations makes it difficult to achieve and retain high quality communication about medications. The participants pointed out that newly employed HCPs receive an inadequate introduction to the discharge process and associated routines.

*“There’s a high staff turnover at the hospital, they don’t get an adequate introduction, the number of temporary nursing staff does not make pleasant reading /…/ These are people who are expected to do a good job. They usually get a three-day introduction. A permanently employed nurse usually gets a three-to-four-week introduction. And they’re [the temporary nursing staff] also very much involved in the medication part of the discharge process.”* (Home care nurse 1, female, focus group 1)

The participants who in their practice had been involved with a discharge nurse perceived the new role as positive and facilitating the discharge process. Some hospital participants expressed the necessity of having a discharge coordinator on the ward to ensure safe discharge. Otherwise, the regular ward nurse responsible for the patient would have to coordinate everything on the day of discharge, resulting in stress and inadequate patient care.

*“And it’s really nice for us nurses to have that back-up from the discharge nurses, because then we have a lot more time with the patients. And maybe even have time to say goodbye when they’re discharged, because you’re not on the phone or writing some patient discharge summary.”* (Hospital nurse 2, female, focus group 3)

## Discussion

This focus group and interview study contributes understanding of communication in general and medication information transfer in particular at hospital discharge of older patients from the perspective of HCPs across healthcare organisations. Three main themes were identified: *Support systems that help and hinder*, *Communication between two separate worlds*, and *The large number of medically complex patients disrupts the communication*. Overall, the findings showed that HCPs viewed the communication at discharge as extremely complex, with a variety of external factors beyond their control affecting the process. This may lead to flawed communication between senders and receivers, making it impossible to adapt the process to the specific patient they are caring for. The fragmented and overworked healthcare system, with many actors from different healthcare organisations, seems to result in communication deficits between senders and receivers at discharge. This induces a feeling amongst HCPs that they are unable to fulfil their professional obligations to the patients.

### The pressure on the healthcare system

The main theme *The large number of medically complex patients disrupts the communication* depicts highly pressurised HCPs caused by a healthcare system that is not adapted to the complexity of caring for older patients. The high patient inflow to hospitals combined with a decreasing number of hospital beds and a greater patient load on primary care with only a minimal increase in resources, constitutes the main obstacle to adequate care of older patients at discharge [35, 46]. The decrease in hospital beds is particularly apparent in Sweden, which has the lowest number of hospital beds per capita amongst comparable nations [47]. This is probably the main reason behind the hospital-based HCPs’ sense of rushed discharges to free up beds for newly admitted patients, which can backfire on the hospitals in the form of a greater risk of readmissions [46]. As further described in this theme, the severe pressure on hospitals is also the reason for HCPs’ difficulties in adapting the discharge communication to the needs of each individual patient. The discharge consultations had to be performed when it was convenient for the HCPs and not the patient. The timing and content of discharge consultations are known to be poorly adapted to patients’ post discharge self-management needs [29, 48, 49]. The importance of written discharge information and informal caregiver involvement for supporting patient information retention was emphasised by the participants. However, the written medication information given to patients is deficient and known to cause confusion among patients and informal caregivers [26, 27], while the HCPs’ inability to adapt the discharge process to the patient prevents the sufficient involvement of informal caregivers [49]. There is therefore a need to find ways of increasing the quality of the discharge information and involving informal caregivers in the discharge process.

Another consequence of the stressful situation is high staff turnover, also mentioned in this theme. This factor inhibits optimal communication between HCPs, as an understanding of each other’s roles and insights in the standardised discharge processes is important for collaboration [33, 36]. Baxter et al*.* have shown that well-established relationships within the healthcare team are essential for facilitating safe hospital discharge [36]. Bringing together HCPs from different parts of the system and providing regular training in standardised discharge procedures have been proposed as solutions [34, 36].

### A fragmented system resulting in unclear responsibilities

The main themes *Support systems that help and hinder* and *Communication between two separate worlds* illustrated the pitfalls of a fragmented healthcare system with numerous healthcare organisations involved. The HCPs reported difficulties navigating the system, which inhibited communication between them. These findings are well in line with previous studies [34, 35, 37]. The many healthcare organisations involved has resulted in the strict regulations for dividing responsibility for a patient, in order to make a certain healthcare organisation accountable and thus, in theory at least, also safer for the patient. However, in reality the regulations have created a system where healthcare units act as separate entities when communicating at discharge, sometimes resulting in unclear responsibilities for patients. Although a clear description of a specific HCP’s responsibility may be stated in the regulations, in reality her/his responsibility may differ. This problem is illustrated in the sub-theme *In between and nowhere – short-term nursing homes as a pitfall*, where follow-up requests sometimes fall between the cracks. This discrepancy between theory and reality in terms of discharge collaboration was previously described by Glette et al*.* [46]. The discrepancy originates from insufficient adaptation of the healthcare system, such as inadequate communication tools and lack of time for collaboration [46]. This means that the healthcare system does not fulfil the regulatory requirements to provide adequate care for older patients at discharge. The creation of the discharge nurse’s role on both the sending and the receiving side is one adaptation of the system that has been introduced. Although the adaptation is viewed as facilitating communication between HCPs in different healthcare organisations, our findings show that it is not enough to bridge the communication gap.

### HCPs’ moral distress connected to their coping strategies

Three different coping strategies used by the HCPs to deal with the discharge situation were revealed in the sub-theme *Strategies for coping with the discharge situation*. We believe that the reason HCPs employ these strategies is to reduce the moral distress caused by the conflicting interests of stakeholders involved in the discharge process. This conflict of interest is caused when the rigid rules and routines do not take the best interest of the patient into account. Moral distress is defined as negative stress symptoms that occur due to ethical situations, where the HCPs cannot protect all interests and values at stake [50]. Irrespective of which coping strategy the HCPs apply in a certain situation, there is a risk of generating moral distress. By applying the first strategy (Smoothening the process by moderating the patients’ autonomy and self-care decisions), both requirements **“**discharging a patient in a timely manner**”** (obligation imposed by the system) and **“**the feeling of the patient being discharged safely**”** (professional obligation) are fulfilled. However, providing opportunities for autonomy is also a professional obligation and an important healthcare outcome goal for older patients [51]. When the third strategy (Adhering to the rules and official routines and accepting that discharge may fail) is applied, the patient’s interests are at risk of being compromised. However, the moral distress is handled by referring to the rules and routines or to another HCP in the discharge process. In these two coping strategies, patient needs become secondary, which is worrying, as there is consensus that a patient-centred care approach should be maintained [52–54]. The HCPs relying on the second strategy (Bypassing the rules and official routines) may fulfil their professional responsibilities, but risk moral distress as a result of deviating from the rules, routines and urgent work responsibilities due to lack of space in the system for them to follow this strategy. Moral distress is known to contribute to the intention to resign, burnout and reduced professional quality of life amongst HCPs [55–57]. It is therefore important in future studies to highlight the problems HCPs experience at discharge in an already heavily strained and short-staffed healthcare system.

### The problematic one-way communication about medications

We believe that the difficulties in communication between HCPs, especially regarding medications as described in the main theme *Communication between two separate worlds,* are due to the one-way communication methods used (discharge summaries, electronic referrals with follow-up requests and the updated medication list). The GPs were especially exposed to the communication gap, as it was apparent that they had strong doubts about the correctness of the information and that it would be transferred to the receivers in a timely manner. The doubts are supported by the knowledge that discharge information transferred from hospitals is often deficient [21, 22, 25, 58]. This is worrying, as impaired transfers of discharge information have been linked to patient safety risks [24]. The GPs’ concerns correspond with previous research on discharge communication including HCPs on both the sending and the receiving side [34, 35, 37]. However, in contrast to previous research, our study was performed in the context of a shared EHR between the hospital and primary healthcare centres. In previous studies [35, 37, 59] a shared EHR was proposed as a possible solution to the communication problems experienced during discharge. Although we believe that the problematic communication gap is probably diminished to some extent with a shared EHR, several of the problems pertaining to correctness of information and delayed discharge summaries still seem to be present.

One solution to the problematic communication gap, which nurses frequently mentioned, is the use of bidirectional communication by means of the phone or a range of IT-systems in addition to the notes in the EHR, despite the fact that the rules and routines state that only written communication should be used. However, there may be challenges in implementing such communication practice amongst physicians in the highly slimmed-down care organisations, as shown by Enzinger et al. [60]. These authors evaluated the feasibility of introducing a phone call between the hospital physicians and GPs prior to discharge. Such phone calls were only successful in 57% of the patients, the main obstacle being the availability of the physicians on both sides. There is therefore a need to find sustainable ways of bidirectional communication between HCPs before implementing such a process.

Another solution would be to include hospital pharmacists more systematically in the discharge process, as the participants stated that they improved the trustworthiness of the medication communication. The quality of discharge documentation has been shown to increase when hospital pharmacists review the medications at discharge [61, 62]. Nevertheless, it is not enough for the information to be sent from the hospital to the next HCPs – it must also be processed and acted upon. Some studies reveal that written information transfer cannot be trusted as the only means of ensuring continuity of care [17, 63]. Our findings in the sub-theme *Worrying that patients will drown in the immense amount of information* demonstrated that due to the complexity and sheer amount of information, the challenge for HCPs is to find ways to support patients to filter and grasp the important details at discharge. Hence, involvement of patients and/or informal caregivers has been called for as a support measure to bridge the communication gap at discharge [29, 49, 63].

### Methodological considerations

In order to enhance the transparency and comprehensiveness of our research methodology, this qualitative study was planned and reported in accordance with the Consolidated criteria for reporting qualitative research (COREQ) [64]. Focus groups were chosen as a primary method, as such groups utilise the interaction between participants to generate in-depth knowledge about people’s experiences [65]. The focus group composition with hospital-based HCPs separated from patient receivers was intended to avoid hierarchical obstacles in the discussions [38]. Our initial strategy was to include six participants in each focus group and to conduct two focus groups in each region, with the option to add more focus groups, if considered necessary. However, due to practical considerations, following the completion of the four focus groups, we decided to conduct individual or group interviews with selected HCPs whose perspectives we deemed were lacking in our collected data.

Investigator and analyst triangulation with multiple researchers was used during data collection and analysis [44], which further strengthens the credibility. We acknowledge the potential influence of our research group’s composition on both data collection and analysis. As the data were collected and analysed by researchers with different genders and professional backgrounds, the risk of potential bias in the data collection and analysis was reduced [44]. To proactively address potential sources of bias related to the focus group sessions, a deliberate approach was undertaken. We appointed a female moderator with a social science background to lead the discussions, while a male co-moderator with a healthcare background was assisting with follow-up questions.

The original aim of the study, which was to identify barriers to and facilitators of transfer of medication information at hospital discharge and how to overcome the barriers, was narrower than the present one. After the first focus group the need for a wider scope became obvious as the participants’ discussions were much broader than expected, making it necessary to also broaden the aim. We discovered that it was difficult to study the barriers to and facilitators in isolation, and the inclusion of the HCPs’ perspective on the communication in general at hospital discharge of older patients provided a more comprehensive picture. No changes were made to the interview guide, as it was open enough to allow for this change of focus.

A limitation of this study was the uneven gender distribution among the participants. Despite our efforts to achieve a more balanced distribution, there was a predominance of women, likely reflecting the gender demographics in these settings. Another limitation was that one of the focus groups consisted of only three participants due to urgent work responsibilities at the hospital. The small group size negatively affected the dynamics and richness of this session. However, a subsequent semi-structured interview was held with two of the absent participants to supplement the data derived from this focus group session. We followed the concept of information power [66] to guide us in the decision about sample size, as it agrees well with the qualitative research paradigm. The widening of our study aim would have required a larger sample. However, our study design with purposeful sampling to identify participants with a wide variation in experience and knowledge, in addition to the use of semi-structured interview guides, improved the quality of the dialogues with the participants, thus increasing the information power and reducing the risk of missing relevant aspects in fulfilling the research aim [66].

The data collection occurred during the Covid-19 pandemic when the healthcare system was under significant strain, potentially affecting the transferability of our findings to different time periods. However, the participants did not indicate any increased communication complexities due to the pandemic. In addition, the pressure on the healthcare system was substantial before and after the pandemic.

## Conclusions

This qualitative study with HCPs across different healthcare organisations showed that communication at hospital discharge of older patients is complex. The fragmentation of the healthcare system has necessitated the use of support systems in the discharge process which sometimes impair communication. It is further limited by the high pressure on the system. The coping strategies employed by HCPs to handle communication difficulties put them at risk of moral distress. Improved methods of communication at hospital discharge are needed for the sake of both the patients and the HCPs involved.

### Supplementary Information

**Additional file 1.**

## Data Availability

The anonymised transcripts used for analysis in the present study are available from the corresponding author on reasonable request.

## Abbreviations

DRP: Drug-related problem
EHR: Electronic health records
GDPR: General data protection regulation
GP: General practitioner
HCP: Healthcare professional
