# Supplementary material for: The complexities of communication at hospital discharge of older patients: a qualitative study of healthcare professionals’ views
Source: BMC Health Serv Res. 2023 Nov 6;23:1211. doi: 10.1186/s12913-023-10192-5 (PMC10626684; doi:10.1186/s12913-023-10192-5)
Supplement: Supplementary file 1 — Additional file 1. [file 12913_2023_10192_MOESM1_ESM.docx]

**Interview guide focus group – receivers**

**Conversation topic 1: to describe the discharge process and barriers and facilitators**

- What do you associate with hospital discharge of a patient?

*Prompt questions:*

- - Which patient groups can be particularly challenging to discharge?
  - How about the discharge of an older patient?
  - How about the patient's medication treatment at discharge?
  - How about the follow-up of a patient after discharge?
  - How individualised do you perceive that a hospital discharge is?
  - When does your responsibility for the patient begin after a patient is discharged?
- When a discharge progress smoothly - what are the reasons?
- When a discharge did not progress smoothly - what are the reasons?

*Prompt questions:*

- - What do you do when the discharge does not progress smoothly?
  - What are the consequences when the discharge does not progress smoothly?
  - What are the reasons for the discharges not to progress smoothly every time?
- What problems do you think older patients experience when they are discharged from hospital?

*Prompt questions:*

- - What problems do you think may arise for an older patient regarding medications at discharge?
  - What problems do you think may arise for an older patient in terms of post-discharge follow-up?
  - How well informed do you think a patient/informal caregiver is about the patient’s medications after a hospitalisation?

**Conversation topic 2: to develop an improved discharge process**

- What information about the patient's medications is important to receive from the hospital at discharge?

*Prompt questions:*

- - What information about the patient's medications do you not want to receive?
  - How do you want to communicate about medications (what kind of information transfer medium)?
- What information about the patient's medications is important that the hospital has given to the patient/informal caregiver at discharge?

*Prompt question:*

- - What information about the patient's medications do you not want the patient/informal caregiver to receive?
- How can the discharge process be improved in terms of patient medication management?

*Prompt questions:*

- - How can the discharge be more individualised?
  - What do you think would happen if informal caregivers would be more involved in the discharge process?

**Conversation topic 3: propose a preliminary discharge model**

**Present the following preliminary findings from previous conducted study on patients’ experience of the discharge process**:

- *Informal caregivers feel that they are not involved in the actual information at discharge.*
- *Patients are rarely given the opportunity to express what is important to them in terms of medication treatment and they are rarely given the opportunity to influence treatment changes that are performed during their hospitalisation.*
- *Patients are often not aware of what a discharge consultation entails and are unprepared for it, which makes it difficult for them to grasp the information.*

**Suggest primary care physicians to have rounds together with hospital physicians when the patient is hospitalised**

- - Thoughts on working according to this model?
  - What obstacles do you see?
  - What conditions need to be in place?

**Interview guide focus group – senders**

**Conversation topic 1: to describe the discharge process and barriers and facilitators**

- What do you associate with hospital discharge of a patient?

*Prompt questions:*

- - Which patient groups can be particularly challenging to discharge?
  - How about the discharge of an older patient?
  - How about the patient's medication treatment at discharge?
    - What do you do if you identify a patient in need of multidose drug dispensing?
  - How individualised do you perceive that a hospital discharge is?
- What problems do you think older patients experience when they are discharged from hospital?

*Prompt questions:*

- - What problems do you think may arise for an older patient regarding medications at discharge?
  - What problems do you think may arise for an older patient in terms of post-discharge follow-up?
- What are your thoughts about the follow-up of a patient's medication treatment after discharge?

*Prompt questions:*

- - When does the hospital's responsibility end when a patient is discharged?
- How do you imagine the receivers (healthcare professionals) of the information?

*Prompt questions:*

- - How do you adapt the information?
  - How do you know what information the receivers want?
- Do you consider contacting/phoning the receiver (healthcare professionals)?
- What are your feelings about the incoming calls from the receivers (healthcare professionals) that you need to answer?
- When a discharge progress smoothly - what are the reasons?
- When a discharge did not progress smoothly - what are the reasons?

*Prompt questions:*

- - What do you do when the discharge does not progress smoothly?
  - What are the consequences when the discharge does not progress smoothly?
  - What are the reasons for the discharges not to progress smoothly every time?

**Conversation topic 2: to develop an improved discharge process**

- What information about the patient's medications is important to provide to the receiving healthcare provider?

*Prompt questions:*

- - How do you want to communicate about medications (what kind of information transfer medium)?
  - What impact do you want the information you provide to the receiving healthcare providers to have?
- What information about the patient's medications is important to give to the patient/informal caregiver at discharge?

*Prompt questions:*

- - What information about the patient's medications do you not want to give to the patient/informal caregiver?
  - How do you want to communication about medications to the patient/informal caregiver (what kind of information transfer medium)?
  - What impact do you want the information you provide to the patient/informal caregiver to have?
- How can the discharge process be improved in terms of patient medication management?

*Prompt questions:*

- - How can the discharge be more individualised?
  - How can the communication with the receiving healthcare professionals be improved?
  - What do you think would happen if informal caregivers would be more involved in the discharge process?

**Conversation topic 3: propose a preliminary discharge model**

**Present the following preliminary findings from previous conducted study on patients’ experience of the discharge process**:

- *Informal caregivers feel that they are not involved in the actual information at discharge.*
- *Patients are rarely given the opportunity to express what is important to them in terms of medication treatment and they are rarely given the opportunity to influence treatment changes that are performed during their hospitalisation.*
- *Patients are often not aware of what a discharge consultation entails and are unprepared for it, which makes it difficult for them to grasp the information.*

1) **Suggest on preparing the patient for discharge consultations and what this entails. Include the informal caregivers in the consultations. Set a scheduled time for the discharge consultation and clearly inform the patient in advance of what the consultation entails**

- What are your thoughts on working according to this model?
- What obstacles do you see?
- What conditions need to be in place?

2) **Propose to have direct contact with primary care physicians when the patient is hospitalised**

- What are your thoughts on working according to this model?
- What obstacles do you see?
- What conditions need to be in place?

3) **Suggest to have the medical round with the patient in a private room**

- What are your thoughts on working according to this model?
- What obstacles do you see?
- What conditions need to be in place?

**Interview guide semi structured interviews**

- Could you briefly tell us about your professional background: workplace, profession and

professional role?

- What do you associate with hospital discharge of a patient?
  - Which patient groups can be particularly challenging to discharge/receive?
    - Prompt: Older patients?
  - How about the patient's medication treatment at discharge?
- *(Question only for short-term nursing home HCPs)*: We in the research group find it a bit difficult with the responsibility issue when a patient is cared for at a short-term nursing home. Can you tell me your views on the responsibility issue when patients are cared for at a short-term nursing home?
  - Prompt: When does your responsibility for the patient end after a patient leaves the short-term nursing home?
- What are your thoughts about the follow-up of a patient's medication treatment after hospital discharge?
  - Prompt: e.g., medication changes performed during hospitalisation?
- *(Question only for short-term nursing home HCPs)*: What are your thoughts about follow-up of a patient's medication when the patient leaves the short-term nursing home?
  - Prompt: e.g., medication changes performed during patient’s stay at the short-term nursing home?
- When you discharge/receive a patient from hospital and the discharge did not progress smoothly – what did not work?
  - Prompt: What problems do you experience related to medications?
  - Prompt: What problems do you experience related to the follow-up of medications?
  - What do you do when you encounter these problems?
  - What are the reasons for discharges not to progress smoothly every time?
- What information about the patient's medications is important to receive from the hospital at discharge?
  - Is there any information you don't want?
  - How do you want to communicate about medications (what kind of information transfer medium)?
- What information about the patient's medications is important that the hospital has given to the patient/informal caregiver at discharge?
  - What information about the patient's medications do you not want the patient/informal caregiver to receive?
- If a hospital discharge is individualised to a patient, how do you notice it as a receiver?
  - Prompt: Can you give examples of individualisation of the discharge process?
- *(Question only for short-term nursing home HCPs)*: When a patient is leaving the short-term nursing home, how do you communicate with any receivers (municipality and primary care centres) of the patient?
  - Prompt: Is there any formalised information transfer?
    - If no: what are your thoughts on this?
    - If yes, what information about the patient's medications is important to provide to the receiving healthcare provider?
    - If yes, what impact do you want the information you provide to the receiving healthcare providers to have?
  - How do you want to communicate about medications with the receiving healthcare provider (what kind of information transfer medium)?
- *(Question only for short-term nursing home HCPs)*: when a patient is leaving the short-term nursing home, how do you communicate with the patient/informal caregiver regarding medication?
  - What information about the patient's medications is important to give to the patient/informal caregiver when the patient leaves?
  - Is there information about the patient’s that you do not want to give to the patient/informal caregiver?
  - What impact do you want the information you provide to the patient/informal caregiver to have?
  - How do you want to communicate about medications with the receiving patient/informal caregiver (what kind of information transfer medium)?
- How can the hospital discharge process be improved in terms of patient medication management?
  - How can the discharge be made more individualised?
  - How can communication with receiving healthcare professionals be improved?
  - What are your thoughts on involving informal caregivers in the discharge process?

***(Questions intended for the senders only)***

**Present the following preliminary findings from previous conducted study on patients’ experience of the discharge process**:

- *Informal caregivers feel that they are not involved in the actual information at discharge.*
- *Patients are rarely given the opportunity to express what is important to them in terms of medication treatment and they are rarely given the opportunity to influence treatment changes that are performed during their hospitalisation.*
- *Patients are often not aware of what a discharge consultation entails and are unprepared for it, which makes it difficult for them to grasp the information.*

1) **Suggest on preparing the patient for discharge consultations and what this entails. Include the informal caregivers in the consultations. Set a scheduled time for the discharge consultation and clearly inform the patient in advance of what the consultation entails**

- What are your thoughts on working according to this model?
- What obstacles do you see?
- What conditions need to be in place?

2) **Propose to have direct contact with primary care physicians when the patient is hospitalised**

- What are your thoughts on working according to this model?
- What obstacles do you see?
- What conditions need to be in place?

3) **Suggest to have the medical round with the patient in a private room**

- What are your thoughts on working according to this model?
- What obstacles do you see?
- What conditions need to be in place?
- Is there anything you would like to add to this interview that you think we have discussed?
- Can we contact you later if something comes up that we want to know more about?
